# Comparative Transcriptome Analysis of Artificially Induced Rough-Mutant *Brucella* Strain RM57 and Its Parent Strain *Brucella melitensis* M1981

**DOI:** 10.3389/fvets.2019.00459

**Published:** 2020-01-10

**Authors:** Xiaowei Peng, Yufu Liu, Yuming Qin, Hui Jiang, Yu Feng, Jiali Sun, Kai Niu, Qiang Gao, Hao Dong, Jiabo Ding

**Affiliations:** ^1^National Reference Laboratory for Animal Brucellosis, China Institute of Veterinary Drug Control, Beijing, China; ^2^South China Agricultural University, Guangzhou, China; ^3^Zhaoqing Institute of Biotechnology Co., Ltd, Zhaoqing, China; ^4^China Animal Disease Control Center, Beijing, China

**Keywords:** transcriptome analysis, stress response, *Brucella*, gene expression, virulence

## Abstract

Brucellosis is one of the most common zoonotic epidemics with a serious threat to public health and livestock development in many countries across the world. Vaccination is a key control strategy toward preventing brucellosis in high-prevalence regions. Recently, a rough-type *Brucella melitensis* mutant strain (RM57) induced from a *B. melitensis* strain M1981 showed protective effects in guinea pigs indicating that it is a good vaccine candidate. In this study, stress response assays were performed to reveal the mechanisms underlying virulence attenuation of RM57. In addition, a genome-wide transcriptome profile of RM57 was analyzed relative to the parent strain M1981 in order to reveal genetic factors controlling the phenotypes. Our results indicated a similar sensitivity to various stress conditions in RM57 owing to a lack of significant differences from its parent strain. Transcriptome analysis showed that a total of 1,205 genes were differentially expressed between RM57 and M1981 with gene ontology terms revealing that these genes are involved in energy production and conversion, translation, ribosomal structure, and biogenesis. Pathway enrichment analysis revealed that genes involved in oxidative phosphorylation, ribosome, nitrogen metabolism, tyrosine metabolism, and two-component system were significantly affected. As a result of these differences at the molecular level, the function of type IV secretion system in RM57 was found to be affected leading to reduced virulence of the RM57 mutant strain in both macrophage and mice infection models.

## Introduction

Brucellosis, an infectious disease caused by *Brucella* spp. and spreads through unpasteurized dairy products, remains one of the most serious bacterial zoonosis across the world. In animals, this pathogen mainly causes abortion in females and male infertility although various other clinical symptoms including fever, night sweats, anorexia, polyarthritis, meningitis, and pneumonia have been reported in humans. Humans usually are infected by *Brucella* spp. through consumption of contaminated dairy products, inhalation of bacterial aerosols as well as contact with wounds or mucosal tissues (1). Due to the serious economic losses and public health risks caused by this disease, regulatory authorities across many countries are engaged in measures toward prevention, control, and elimination of brucellosis in domestic animals thereby guaranteeing safety of dairy products (2).

So far, vaccination has been successful as a control strategy for preventing exposure to brucellosis in regions where the disease is prevalent (3). Consequently, various studies have demonstrated that live *Brucella* vaccines such as S19, RB51, S2, and Rev.1 are able to effectively offer immune protection against brucellosis and these are therefore widely used around the world (4). However, negative implications of these vaccines including diagnostic interference, residual virulence in the host, and pathogenic effects to human health cast doubts to this control strategy (3, 5–7).

To overcome these drawbacks and build an efficient and reliable vaccine strategy against the disease, a rough attenuated vaccine candidate *Brucella melitensis* RM57 (Accession: SAMN12827002), free of antibiotic resistance, was developed from its parent strain *B. melitensis* M1981 (Accession: SAMN12827001). As RM57 was attenuated in mice and guinea pig infection models and showed promise as an ideal vaccine candidate, we aimed to unravel the mechanism of virulence attenuation of the RM57 strain through phenotypic characterization and transcriptome analysis. In this study, we therefore assessed RM57 phenotypes that are pertinent to infection and virulence using macrophages and mice models and further performed RNA sequencing analysis to study the genetic factors controlling the observed phenotypes.

## Materials and Methods

### Ethics Statement

The female BALB/c mice were handled in strict accordance with the Experimental Animal Regulation Ordinances defined by the China National Science and Technology Commission. The study was approved by the animal ethics committee of China Institute of Veterinary Drug Control under permit number (CIVDC 2019-000672).

### Bacteria Strains and Culture Conditions

The *B. melitensis* M1981 strain was isolated in China in 2007. For induction of the RM57 rough strain, the M1981 strain was first induced with tryptic soy broth (TSB, Becton, Dickinson and Company, Sparks, MD, USA) medium containing chloramphenicol (1 μg/ml) for 40 generations. The rough-type colony was selected and incubated with anti-*B. melitensis* serum at 37°C for 2 h and 4°C for 12 h. The rough-type colony of the non-agglutinated bacteria culture medium was selected and induced with anti-*B. melitensis* serum again as described above. After the rough-type strain was induced by the antiserum for 45 generations, it was continuously passaged 12 generations in guinea pigs to finally obtain the RM57 strain, which was stable in both phenotype and bacteria virulence.

The *Brucella* strains were routinely grown in TSB at 37°C or on tryptic soy agar (TSA, Becton, Dickinson and Company, Sparks, MD, USA) medium incubated at 37°C. All the bacterial strains were frozen at −80°C and supplemented with 25% (v/v) glycerol.

For the growth curve assay, *Brucella* strains RM57 and M1981 (initial density of 1 × 10^6^ CFU/ml) were grown in TSB medium at 37°C with continuous shaking. The CFUs of RM57 and M1981 were measured by plate count at different time points.

### Lipopolysaccharide Extraction and Silver Staining

The parent strain M1981 and rough type strain RM57 were cultured in TSB medium then grown to the stationary phase at 37°C with continuous shaking. Bacterial cells were collected by centrifugation then LPS was extracted using an LPS Extraction Kit (iNtRON, Seoul, Korea) following the manufacturer's instructions. Samples were loaded on a 12% polyacrylamide gel for SDS-PAGE analysis followed by the silver staining assay as previously described (8).

### Transcriptome Analysis

To understand genetic regulation of virulence in the *Brucella* strains under this study, we performed a transcriptome analysis for M1981 and RM57. Briefly, 3 samples of each *Brucella* strain from a single colony was grown in tubes with TSB at 37°C until the log phase was reached. Total RNA was then isolated from the bacterial pellets using the TRIzol method (Invitrogen, Carlsbad, CA, USA) according to the manufacturer's instructions. Residual DNA in the RNA samples was removed using DNase I (Thermo Scientificm, USA). RNA concentration and purity were determined using a nanodrop spectrophotometer (ND 1000 spectrophotometer Thermo Scientific, Wilmington, USA).

The sequencing library of each RNA sample was prepared by using NEBNext Ultra Directional RNA Library Prep Kit for Illumina as recommended by the manufacturer. In brief, RNA fragments were reverse-transcribed and amplified to double-stranded cDNA and then ligated with an adaptor. The amplified cDNA was purified using magnetic bead based method, and the molar concentration was determined for each cDNA library. The HiSeq 4000 platform was used to perform the transcriptome sequencing. Sequencing and subsequent bioinformatics analysis were completed at Novel Bioinformatics Co., Ltd, Shanghai, China.

### Quantitative Real Time Polymerase Chain Reaction

To validate gene expression, a quantitative real time polymerase chain reaction (qRT-PCR) was performed as previously described (9) targeting differentially expressed genes earlier identified in the RNAseq analysis. The genes targeted as well as primer sequences used for qRT-qPCR are provided in Table S1. Real time PCR was run in triplicates in a 20 μl reaction volume containing 10 μl 2×SYBR® Premix Ex TaqTM II (TAKARA), 100 nM forward and reverse primers, and 1 μl appropriately diluted cDNA as the template. To normalize the expression, 16S rRNA, which is constantly transcribed in bacteria, was used as an internal amplification control. qRT-PCR analysis was performed in an Applied Biosystems 7500 Real-Time PCR System and the relative transcription levels were determined by the 2^−ΔΔCt^ method.

### Determination of Cell Viability

To investigate intracellular viability of M1981 and RM57 strains *in vitro*, we obtained RAW264.7 cells from the Cell Resource Center, IBMS, CAMS/PUMC (Beijing, China) and performed cell infection assays as previously described (10). Briefly, cells were cultured in 24-well plates (Corning, NY, USA) then infected with *Brucella* strains at a multiplicity of infection (MOI) of 100. After 1 h of incubation, plates containing the cells were washed three times using PBS and then incubated in a medium containing gentamycin (50 μg/ml) to kill extracellular bacteria. At 1, 12, 24, 48, and 72 h post-infection (hpi), the cultures were washed and lysed with 500 μl 0.1% (v/v) Triton X-100-water solution, then the number of surviving bacterial cells was determined using the plating technique on TSA. The experiment was performed in triplicates and repeated at least twice.

### Infection Assays Using Animal Models

A total of 45 female 4–6 week-old BALB/c mice were used in the infection assay. The test animals were acquired from the Beijing Vital River Laboratory Animal Technology Co., Ltd then randomly divided into three groups. In each group, mice were inoculated intraperitoneally with 100 μl (~1 × 10^5^ CFUs per animal) of RM57 mutant strain and the parental strain M1981. For the control group, PBS solution was used. Infected and control animals (*n* = 5 per group) were euthanized at 14, 28, and 42 days post infection (dpi) via carbon dioxide asphyxiation, sacrificed then their spleens analyzed for level of infection. First, the spleen was carefully detached from the rest of the body, weighed and homogenized in 1 ml of PBS. Bacterial colonies in each sample were then enumerated by TSA plate count followed by calculation of CFUs per spleen.

### Sensitivity of *Brucella* Strains to the Bactericidal Action of Non-immune Serum

We evaluated the effect of bactericidal action in non-immune serum on sensitivity in *Brucella* strains according to a previously reported protocol (11). Briefly, cultures of RM57 and M1981 strains were adjusted to 10^4^ CFUs/ml using PBS then dispensed in microtiter plates (at a volume of 30 μl/well) containing 60 μl of new-born bovine serum. Cultures were incubated for 90 min at 37°C with gentle agitation then complement action was blocked by adding 150 μl/well of TSB medium. The TSB medium was mixed well with the bacterial suspension then 75 μl was plated on TSA plates in triplicates. The plated cultures were incubated at 37°C for 3 days followed by an analysis of sensitivity. The results were expressed as the percentage of CFU recovered with respect to control samples where new-born bovine serum was substituted by PBS.

### Determination of *Brucella* Strain Susceptibility Under Stress Conditions

To test susceptibility of *Brucella* strains under stress conditions, an assay was modified from a previously reported study (10).

In stress assay, initially both *Brucella* strains (with an initial density of 1 × 10^7^ CFU/ml) were cultured in TSB (5% EtOH, pH4.5, 2.5 mM H_2_O_2_) for 2 h. Afterwards, the concentration of bacteria was measured by plate count, and the survival rate (%) was calculated as surviving bacteria relative to the TSB control.

In order to determine the sensitivity of RM57 mutant to heat shock stress, iron deficiency and hypertonic environments, the bacteria were cultured in TSB medium and treated with 42°C (heat shock stress), 10 mM 2′2- dipyridyl (iron deficiency), and 500 mM NaCl (hypertonic environment), respectively. The bacteria were cultured in this medium at the same initial density (1 × 10^7^ CFU/ml) and the CFUs were determined at 24 and 48 h post-treatment for both strains. The survival rate (%) was calculated as surviving bacteria relative to the 37°C as optimum temperature control.

### Effector Translocation Assay

To determine whether the type IV secretion function in RM57 strain was affected, an effector translocation assay was performed as previously described (12, 13). Briefly, a TEM1-VceC fusion plasmid based on the original pZL1790-TME1 was constructed then introduced into M1981 and RM57 by electroporation. To perform the effector translocation assay, 5 × 10^4^ RAW264.7 macrophages were seeded into 96-well plates and infected with M1981 and RM57 expressing the TEM1-VceC fusion protein at a multiplicity of infection of 1,000:1. Cells were incubated for 30 min at 37°C in 5% CO_2_ then each plate washed twice with PBS and 0.1 ml DMEM with 0.5 mM IPTG. The plates were incubated at 37°C in 5% CO_2_, washed once with Hank's balanced salt solution (Gibco, Grand Island, NY) 15 h post-infection then loaded with a solution containing the fluorescent substrate CCF4/AM at a final concentration of 1 mM. The cultures were left to stand for 2 h at room temperature according to the standard protocol (Invitrogen, Carlsbad, CA) then the translocation of TEM1-VceC fusion protein into the macrophages was analyzed by fluorescence microscopy.

### Statistical Analysis

Basic statistical analyses were performed using SPSS 16.0 (SPSS Inc., Chicago, IL, USA). In the virulence assay of RM57 strain in mice, an unpaired Student's *t*-test was performed at each time point. In the group analysis, Analysis of Variance (ANOVA) method was used for data analyses. Differences were considered significant at *P*-values of <0.05.

## Results

### Growth and Lipopolysaccharide (LPS) Characterization of RM57 Mutant Strain

**Figure 1:**
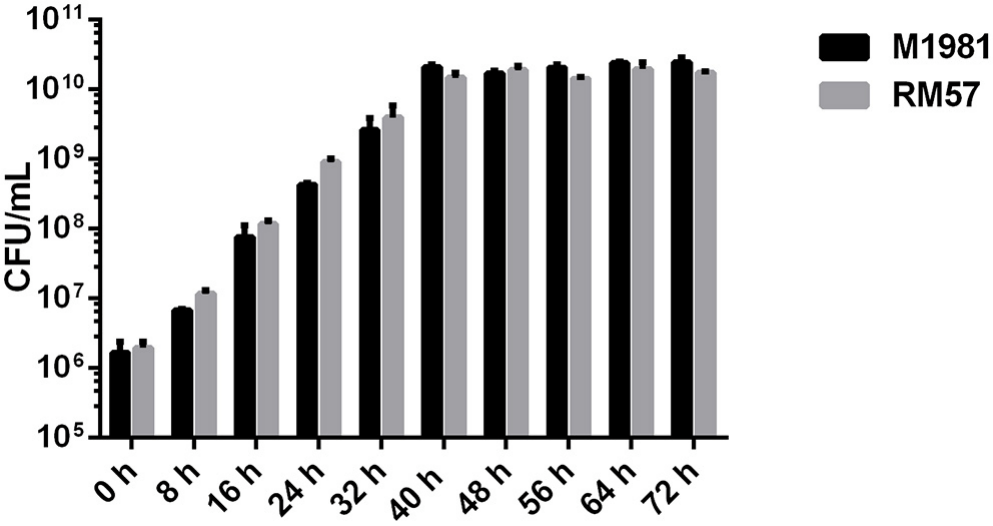
Growth rate of RM57 mutant and its parent strain M1981 in TSB medium. *Brucella* strains (initial density of 1 × 10^6^ CFU/ml) were grown in TSB at 37°C with continuous shaking for 72 h. The CFUs of RM57 and M1981 were measured at different time points.

**Figure 2:**
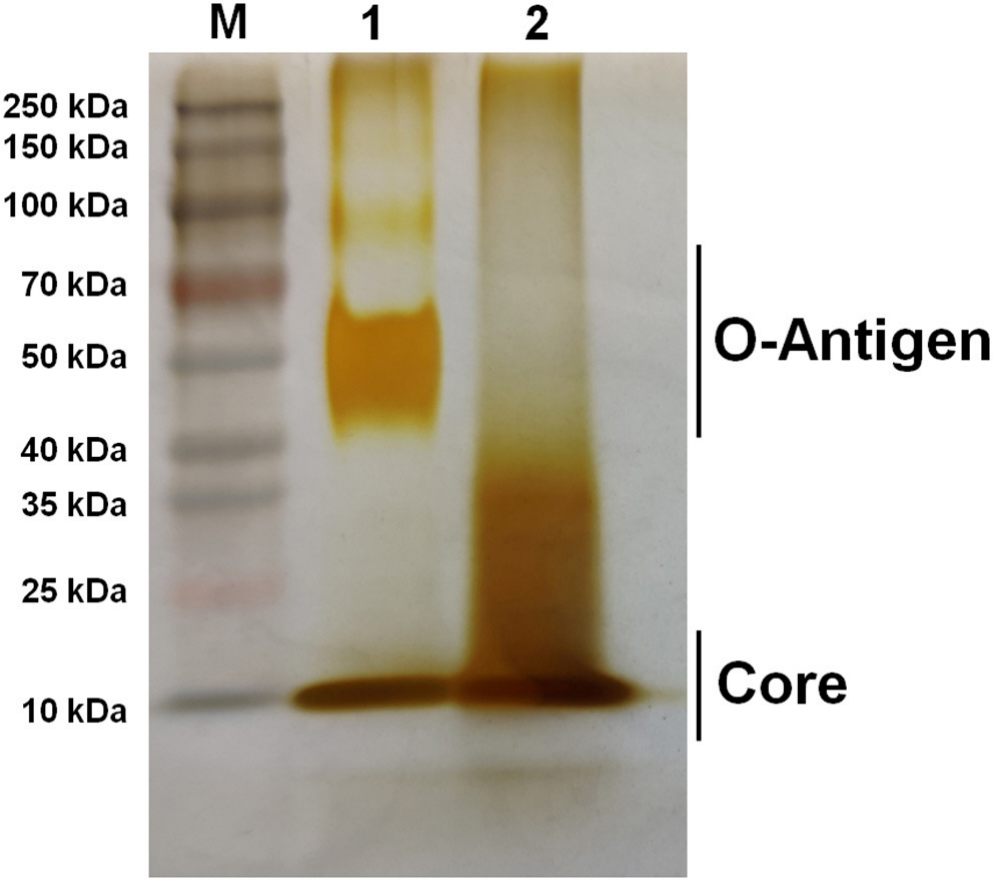
Silver staining of bacterial LPS. Lane M: Prestain page ruler (Thermo Scientific, USA); Lane 1: M1981 LPS; Lane 2: RM57 LPS.

To characterize the growth rate of *Brucella* mutant strains, RM57 and M1981 were cultured in TSB medium at 37°C following a similar initial optical density. We observed that the growth rate of RM57 mutant strain was almost similar as its parent strain M1981 (Figure 1). On the other hand, findings from the LPS silver staining showed that the rough type *Brucella* RM57 had a modified pattern of O-antigen compared to its parent (Figure 2).

### RM57 Was Attenuated in Macrophage and Mice Infection Models

**Figure 3:**
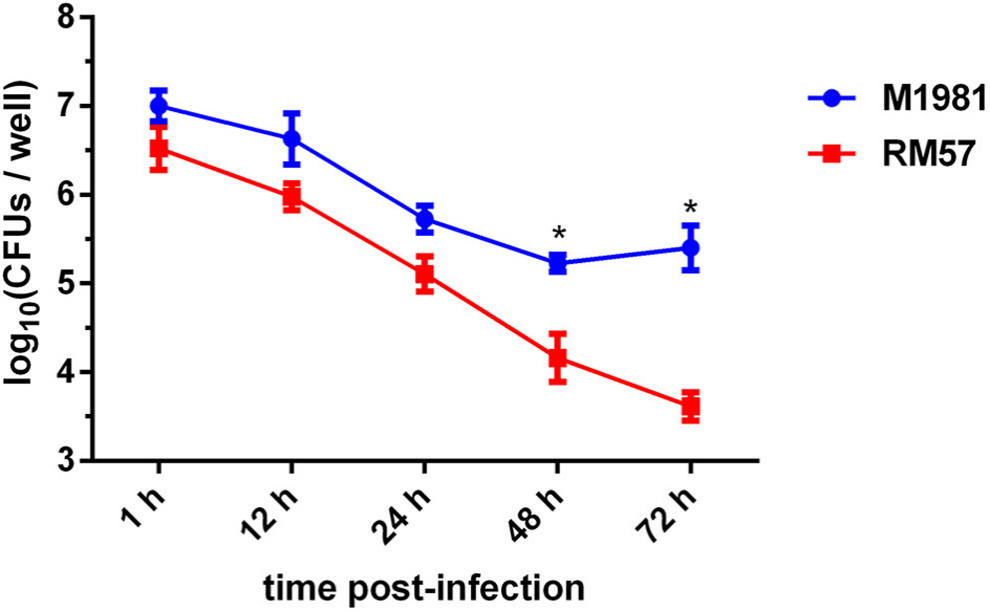
Profiles of virulence attenuation in RM57 in macrophage infection models. Multiplication of RM57 and M1981 strains in RAW264.7 macrophages over 72 h. Values represent the means of three experiments performed in duplicate while error bars indicate the standard deviation (SD) of the mean. *p < 0.05.

To assess virulence of the RM57 strain, its ability to multiply within a cultured macrophage RAW264.7 was tested. It was observed that intracellular bacterial load of the RM57 strain was almost similar to that of M1981 during the first 24 h post infection. However, RM57 load significantly reduced at 48 (*P* < 0.05) and 72 h (*P* < 0.05) in murine macrophages compared to the parent line M1981 (Figure 3).

**Figure 4:**
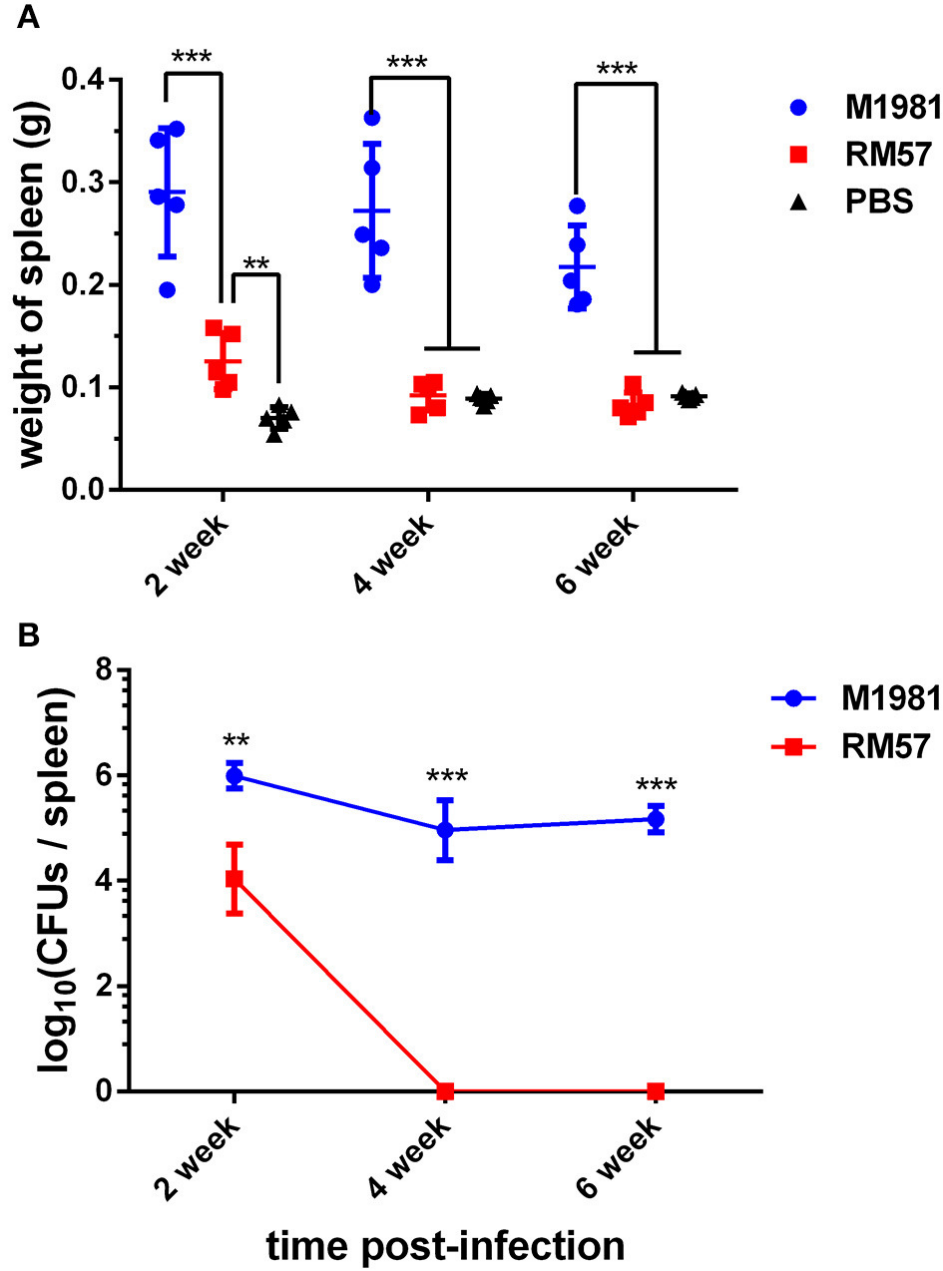
Virulence of RM57 attenuation in mice infection models. **(A)** Spleen weight of RM57 and M1981 infected mice at 2, 4, and 6 weeks post infection. **(B)** Splenic CFUs of RM57 and M1981 infected mice at 2, 4, and 6 weeks post infection. Values represent the means of spleen weight or splenic CFUs at each time point, and the error bars indicate the standard deviation (SD) of the mean. *p < 0.01 and ***p < 0.001.

A similar pattern was observed when mice models were used. Analysis of spleen weight following infection showed that mice infected by the RM57 mutant strain had a significantly lower weight than those infected with M1981 at 2, 4, and 6 weeks post infection (*P* < 0.001). On the other hand, spleens recovered from mice infected with RM57 were significantly heavier than those from the negative control group (*P* < 0.01) at 2 weeks post infection while at 4 and 6 weeks post infection, spleens from mice infected with RM57 and PBS group showed no significant difference (*P* > 0.05) (Figure 4A). In addition, the RM57 mutant recovered from the spleens of mice was significantly reduced at 2 week post infection (*P* < 0.01) with no mutant strain recovered from the spleens of mice at 4 and 6 weeks post infection (Figure 4B).

### RM57 Shows No Sensitivity to Stress Conditions

**Figure 5:**
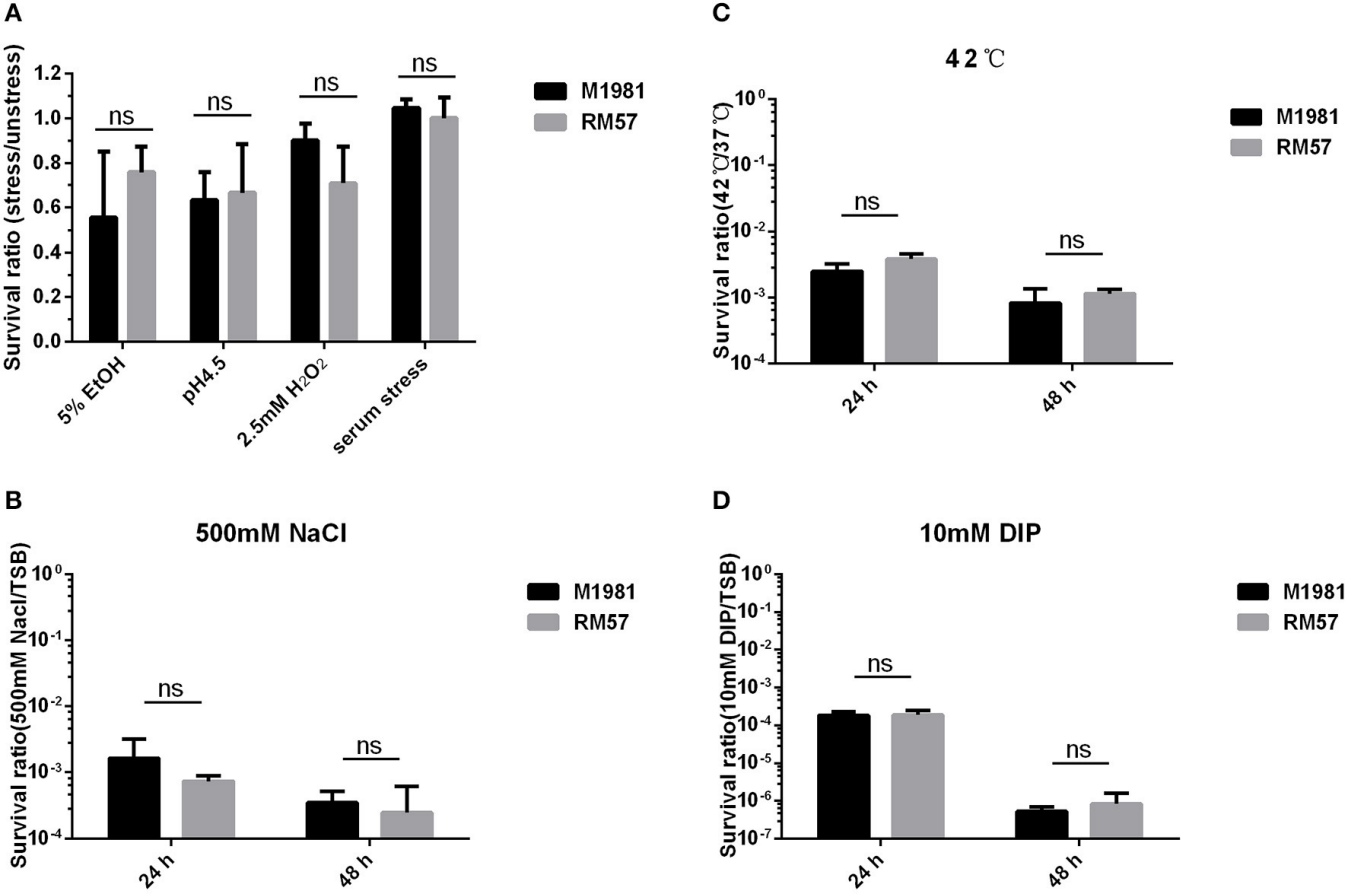
**(A)** The survival ratio of RM57 and M1981 cultured in TSB (5% EtOH, pH4.5, 2.5 mM H_2_O_2_, and non-immune serum) for 2 h. **(B)** The survival ratio of RM57 and M1981 cultured in TSB at 42°C (heat shock stress). **(C)** The survival ratio of RM57 and M1981 cultured in TSB with 500 mM NaCl (hypertonic environment). **(D)** The survival ratio of RM57 and M1981 cultured in TSB with 10 mM 2′2-dipyridyl (iron deficiency).

Assaying sensitivity of *Brucella* strains against a hypertonic environment, iron deficiency medium, and heat stress conditions revealed no significant effect of these conditions to the bacteria. Particularly, survival rate of the RM57 mutant strain showed normal growth in TSB medium as well as when challenged with H_2_O_2_, and 5% EtOH (Figure 5A). Similarly, there was no observable difference in survival ratio of the two strains when challenged with acidic environment (Figure 5A). Previously, the rough type *Brucella* strains have been described as being more sensitive to the bactericidal action of normal serum than their smooth counterparts (11). However, after incubation for 90 min in non-immune serum, we observed that the survival rate of RM57 was not significantly reduced compared to the parent strain M1981 (Figure 5A). Similarly, exposure to the other stress conditions of hypertonic environment, iron deficiency medium and heat stress conditions revealed no differences in survival rates (Figures 5B–D).

### Identification of Differentially Expressed Genes in *Brucella* Strains

**Figure 6:**
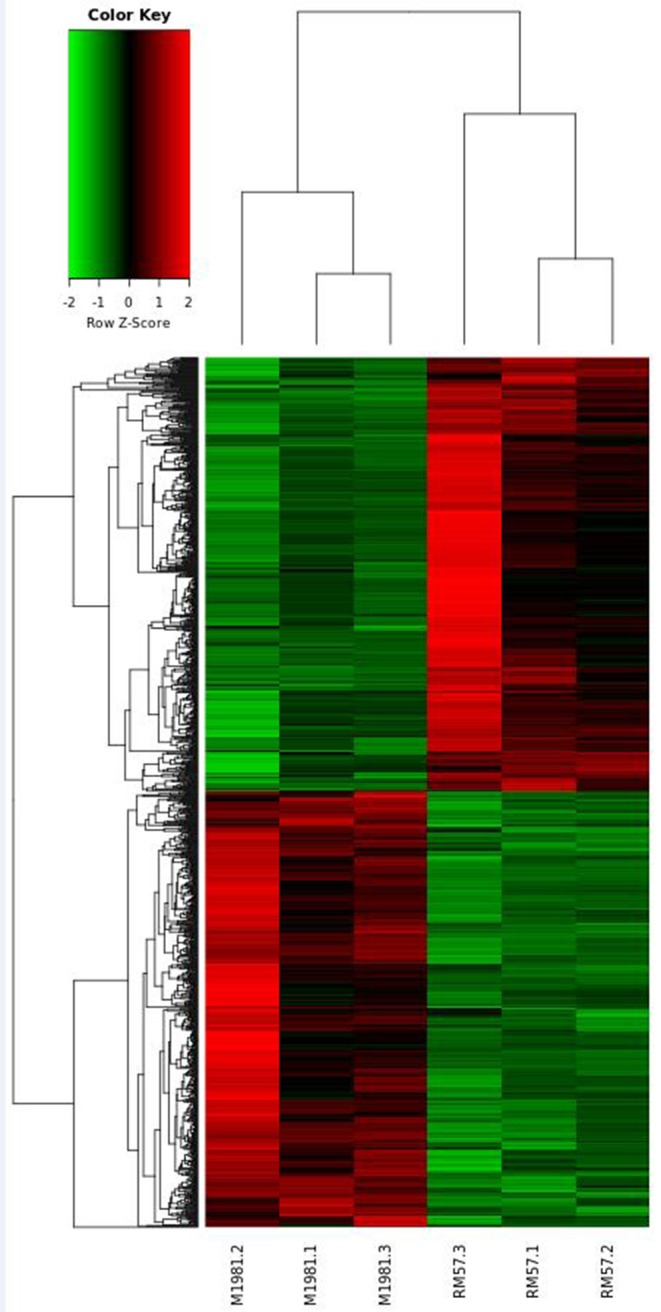
Heat map of the comparative transcriptome analysis of RM57 and M1981.

To reveal genetic factors regulating virulence attenuation of RM57 relative to the parent strain, changes in transcriptional profiles of gene expression in the two strains were determined using transcriptomic analysis following RNA sequencing. The transcriptome dataset has been submitted to the GEO repository and is available using accession number GSE131394. Transcriptome data showed that a total of 1,205 genes exhibited significant differences (log2FC > 1 or <−1 and FDR < 0.05) and among these, 597 were significantly up-regulated while 608 were significantly down-regulated in RM57 compared to its parent strain M1981 (Figure 6). Among these, the highest ranked upregulated genes included BMEI1041, BMEII0642, BMEII1003, BMEI1040, BMEII0987, BMEII0105, BMEI1362, BMEII0988, BMEII0353, and BMEII0321 while BMEI0427, BMEI1313, BMEI0877, BMEII0948, BMEII0759, BMEI0422, BMEI0386, BMEII0758, BMEI0454, and BMEII0844 were highly down-regulated.

**Figure 7:**
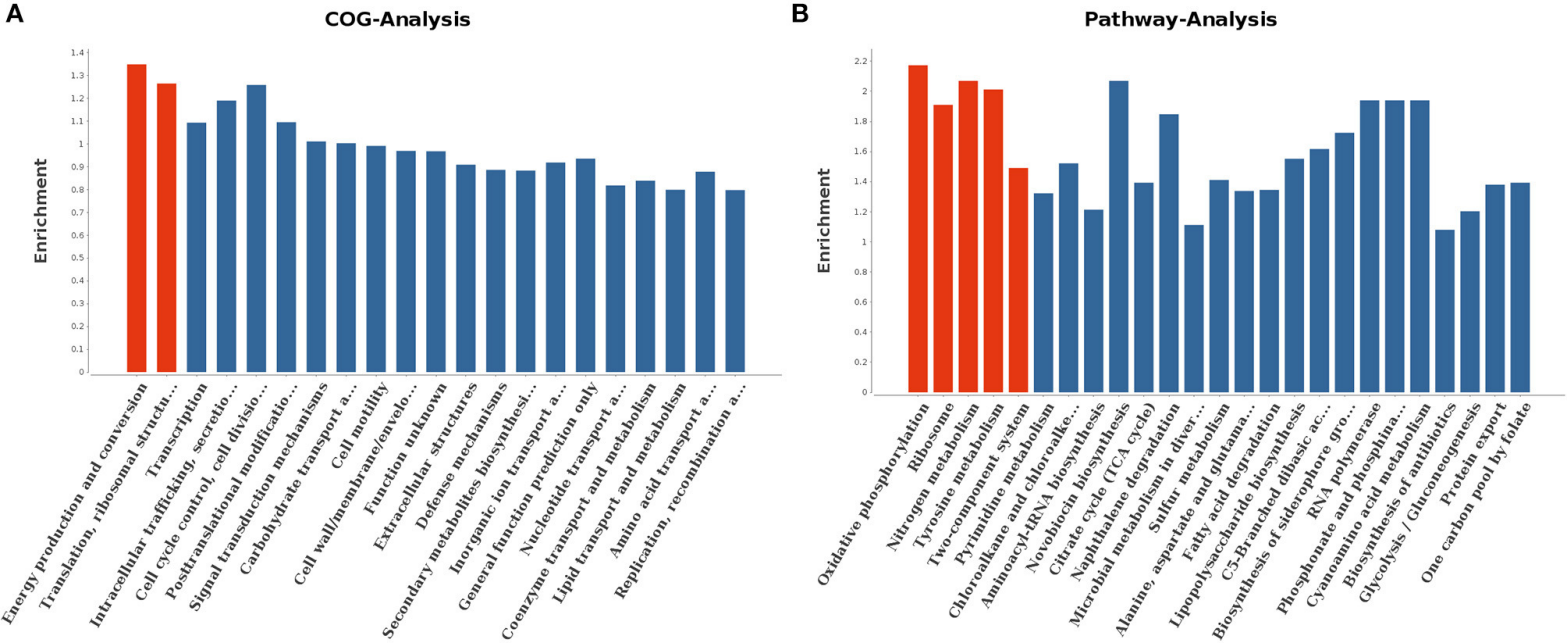
COG analysis **(A)** and KEGG pathway analysis **(B)** for differentially expressed genes. The red columns indicated these categories or pathways were significantly enriched (*p* < 0.05) while the blue columns means these categories or pathways were not significantly enriched.

With regard to gene ontology terms, the differentially expressed genes mainly comprised those involved in energy production and conversion and translation, ribosomal structure and biogenesis (Figure 7A). KEGG pathway analysis further revealed that the genes which exhibited significant differences were primarily enriched in oxidative phosphorylation, ribosome, nitrogen metabolism, tyrosine metabolism, and two-component system (Figure 7B).

To validate the RNA-seq result, 16 genes were chosen and quantified *via* qRT-PCR. Expression levels of the 16 selected genes resulted in a similar profile using both qRT-PCR and RNA-seq and this is outline in Table 1.

**Table 1:** Transcriptional level of 16 selected genes by RNA-seq and RT-qPCR.

**Gene**	**Log**_****2****_ **fold change (M1981 vs. RM57)**		**Function**
	**qPCR**	**RNA-seq**	
BMEI0877	3.05	3.98	Bacterial nucleoid DNA-binding protein
BMEII0759	5.92	3.88	Cytochrome bd-type quinol oxidase, subunit 2
BMEI0454	1.73	3.75	Outer membrane protein W
BMEII0987	−2.41	−4.63	Uncharacterized conserved protein
BMEI1040	−8.93	−4.65	ABC-type transport system involved in Fe-S cluster assembly, permease component
BMEII1003	−5.00	−4.65	Membrane protein involved in the export of O-antigen and teichoic acid
BMEII0642	−2.33	−4.77	Transcriptional regulator
BMEII0949	3.83	3.45	Nitrate reductase alpha subunit
BMEI0632	1.13	3.44	hypothetical membrane spanning protein
BMEII1116	7.73	5.60	LuxR family transcriptional regulator, quorum-sensing system regulator VjbR
BMEI0872	4.71	2.27	host factor-I protein, Hfq
BMEII0704	6.02	8.00	Bacterioferritin (cytochrome b1)
BMEI0569	6.58	7.00	manganese transport protein MntH
BMEII0906	7.36	6.50	acid stress chaperone HdeA
BMEII0581	9.36	4.68	Cu/Zn superoxide dismutase
BMEII0423	4.03	3.90	fructose-bisphosphate aldolase

### Expression Profiles of Genes Encoding Virulence Factors Show a Variation Between the Mutant and Parent Strain

Based on the result of RNA-seq, it was found that the expression levels of several genes encoding virulence factors were significantly reduced in the mutant strain relative to the parent line. Particularly, BMEII1116 (encoding quorum-sensing system regulator VjbR) and BMEI0872 (encoding Hfq protein) were both significantly down-regulated in RM57 relative to M1981. A similar profile was observed in the qRT-PCR result (Table 1). In addition, expression of four genes in the *virB* operon (BMEII0029, BMEII0030, BMEII0034, and BMEII0035) which encodes the type IV secretion system showed a downregulation recording a reduction in the fold change from 2.2 to 3.7 fold.

### Type IV Secretion System Is Affected in RM57 Mutant Strain

**Figure 8:**
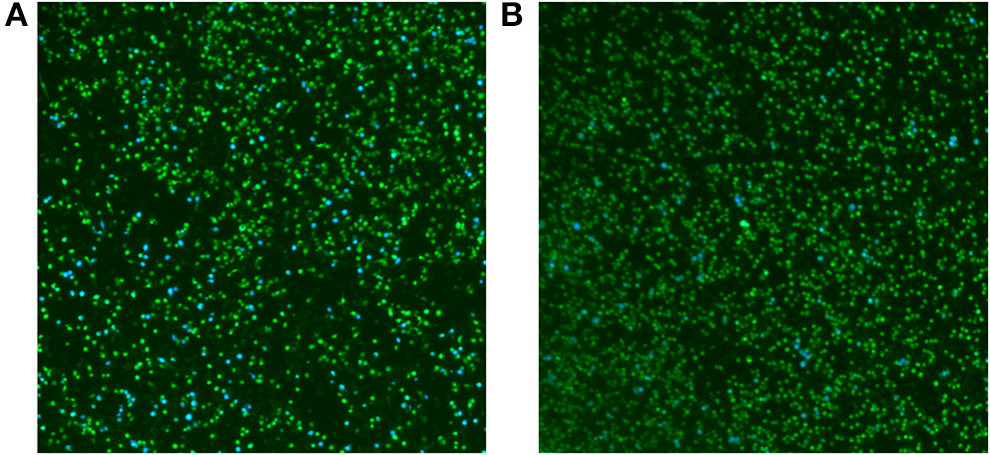
Translocation of the effector VceC into mouse macrophages RAW264.7 infected by M1981 **(A)** and RM57 mutant strain **(B)**. Cells in which translocation of the fusion protein has occurred appear as blue. The result was a representative individual experiment repeated three times.

To determine whether the function of T4SS in RM57 was affected, M1981 and RM57 expressing the TEM1-VceC fusion protein were used to infect RAW264.7 macrophages to determine the translocation of the VceC protein (an effector of the *Brucella* Type IV secretion system translocated into macrophages) (12, 13). We observed the translocation of TEM1-VceC protein into RAW264.7 macrophages infected by M1981, and the percentage of blue cells was ~10.25% at 15 h post-infection (Figure 8A). However, about 1.69% of the cells were blue when infected with RM57 (Figure 8B). These results indicated that the function of T4SS was affected in the RM57 mutant strain.

## Discussion

The current study describes phenotypic characterization and transcriptome analysis of two strains of *B. melitensis* and further reveals sensitivity to various stress conditions. RM57, a rough mutant strain was previously induced from a parent line *B. melitensis* isolate M1981 and has been shown to be attenuated in both mice and guinea pig infection models (14). Results of the current study corroborated this earlier report indicating that RM57 was attenuated in both microphage and mice infection models. Previous studies have demonstrated that LPS of *Brucella* played a key role in virulence as it could impair normal recognition by the innate immune system thereby delaying an organism's immune response (11). According to our LPS silver staining, we observed that O-antigen in RM57 was modified and this was consistent with the increased sensitivity to SDS and polymyxin B in the RM57 mutant strain as previously reported (14). It is therefore possible that the defective LPS was involved in the reduced virulence of RM57 mutant strain relative to the parent line M1981.

Facultative intracellular pathogens, such as *B. melitensis*, encounter considerable environmental stresses during their interactions with host cells including exposure to reactive oxygen and nitrogen species, unfavorable pH ranges as well as nutritional deprivation (15). In line with this, we carried out stress response assays for the *Brucella* mutant strains under induced stresses of heat, iron deficiency, and hypertonic environment and compared the findings with those from the parent line. Our data indicated that RM57 was not sensitive to the aforementioned stress conditions.

To further unravel the genetic factors regulating the related phenotypes and virulence attenuation of RM57 relative to the parental line, we generated a transcriptome profile of RM57 and M1981 and determined differentially expressed genes. We observed that up to 1,205 genes were differentially expressed (>2-fold in RM57) thereby indicating that RM57 exhibits transcriptional changes relative to M1981. Among the differentially expressed genes mutant and the parent, we focused on the down-regulated genes in mutant strain. Here, several genes involved in *Brucella* virulence on a host were significantly down-regulated in the mutant including BMEII1116, BMEI0872, and *virB* operon. The BMEII1116 gene encodes a LuxR-type regulator VjbR which is a well-known virulence factor of *Brucella* spp. with various studies demonstrating the major role played by this gene in modulating expression of several *Brucella* virulence factors such as VirB operon, VirB effectors, flagellar components, and cell surface structures (12, 16).

Similarly, previous transcriptomic and proteomic analyses have implicated VjbR indirect or indirect expression of hundreds of genes (17, 18). In the current study, expression levels of *vjbR* was reduced more than 5-fold, pointing toward a possible reduction in virulence of RM57. In addition, as VjbR could directly up-regulate the expression of *virB* operon in *Brucella* strains, the low-expression level of VjbR might not completely induce the expression of *virB* operon in RM57 (12).

Our results further showed that, expression levels of *hfq* were significantly reduced in the mutant strain relative to the parent. Hfq protein, encoded by the BMEI0872 gene is an important RNA chaperone involved in stress resistance and *Brucella* virulence in both macrophage and mice infection models (19). Furthermore, recent studies have demonstrated that this chaperone can regulate *virB* operon expression and plays a key role in the interaction between sRNAs and their target mRNAs (20, 21). Downregulation of this gene in our mutant strain therefore indicates a reduction in virulence relative to the parental line. We also observed a marked downregulation of type IV secretion system encoding genes in RM57 relative to the parent line. Type IV secretion system is important in *Brucella* spp. for evading fusion of endosome vesicles with lysosomes through secretion of effector protein. This system, which is encoded by *virB* operon, also plays a key role in *Brucella* virulence. *Brucella* mutant strains that lack a functional type IV secretion system are reported to result in high attenuation in both macrophages and mouse infection models (12).

Furthermore, we observed that the function of type IV secretion system in the mutant strain was affected relative to its parent strain thereby indicating that an abnormal type IV secretion system could have been the cause of the observed reduction in bacterial virulence.

In summary, although the mutant *Brucella* strain was not sensitive to various stress responses at the phenotypic level, comparative transcriptome analysis and expression profiles of various important virulence factors revealed changes between the mutant and parent line. These changes at the molecular level could be playing a key role in the attenuation of RM57.

## Conclusion

This work provides insights into the molecular regulation of LPS deficiency in RM57, which was unknown, and further generates knowledge on the differences between this mutant and its parental line.

## Data Availability Statement

The data in this study can be found in the GEO database (https://www.ncbi.nlm.nih.gov/geo/), with the accession number: GSE131394.

## Ethics Statement

The animal study was reviewed and approved by Animal ethics committee of China Institute of Veterinary Drug Control.

## Author Contributions

JD, XP, and HD designed the experiments. YL, HJ, KN, QG, and JS performed the experiments. HD and YQ prepared the manuscript. YF analyzed the data. All authors have read and approved the final version of this manuscript.

### Conflict of Interest

YL was employed by company Zhaoqing Institute of Biotechnology Co., Ltd. The remaining authors declare that the research was conducted in the absence of any commercial or financial relationships that could be construed as a potential conflict of interest.
