# Supplementary material for: Comparative Transcriptome Analysis of Artificially Induced Rough-Mutant Brucella Strain RM57 and Its Parent Strain Brucella melitensis M1981
Source: Front Vet Sci. 2020 Jan 10;6:459. doi: 10.3389/fvets.2019.00459 (PMC6966878; doi:10.3389/fvets.2019.00459)
Supplement: Supplementary file 1 [file Table_1.pdf]

Supplementary Material

Table S1 Primers used in this study

Primers	Sequences (5'to 3')	Used for
16S rRNAf	TACCAGCCCTTGACATCC	RT-qPCR
16S rRNAr	TCATCCCCACCTTCCTCT	
BMEI0877-F	AAAAGGGCGGTTTGACGA	
BMEI0877-R	TTGCGGGCGGGAATAG	
BMEII0759-F	CCTGGTTTGCGAACTATGC	
BMEII0759-R	ACGCCGACAGACGAGATG	
BMEI0454-F	TTATCCTCGGCACGACCTA	
BMEI0454-R	ACGCTTCCCGCATCCT	
BMEII0987-F	AAGTGCCCGTAAGCAGGAT	
BMEII0987-R	TTGCGTCCATTCCCACA	
BMEI1040-F	AGAATACCCGCTCCACCG	
BMEI1040-R	CATCAAACTCGCCTTCATCC	
BMEII1003-F	CGCTCGGGTCCAATGTC	
BMEII1003-R	ATCAACGCAAGGTTCAAGGT	
BMEII0642-F	TCATCCAGCCGCACCTC	
BMEII0642-R	TCATCCAGCCGCACCTC	
BMEII0949-F	ATGGGATGAGGTCAACGAGA	
BMEII0949-R	CGAGCAGCGACAGATAACG	
BMEI0632-F	TATCACGGTGCGAACTGG	
BMEI0632-R	TGGGCGGCATAGGTCTT	
BMEII1116-F	TATGACGCTGCCTGCTACC	
BMEII1116-R	GGATACGGCGATGAAAAGAT	
BMEI0872-F	TCGCAAAATCTACAAGACCTC	
BMEI0872-R	CTTCCTCGCCTTCAAACA	
BMEII0704-F	AACGATTGGGGTTACACG	
BMEII0704-R	GCGAAACGGTCTGGAGG	
BMEI0569-F	AATCGGCGTTATCCTCACT	
BMEI0569-R	AGCATATCGGGGTTGCT	
BMEII0906-F	TCGTGGGTTGGGTCGTA	

BMEII0906-R	GAGTTCTGCTTCAGCCTTTT
BMEII0581-F	AGAAGTTGGCACCGTGGTC
BMEII0581-R	TCAGGCGTGGCAAATCG
BMEII0423-F	ATCACGCCGCTGAAAAG
BMEII0423-R	GAGATGCACGCAGACCG
